# First Record of *Clonostachys rosea* as an Entomopathogenic Fungus of the *Cephus fumipennis* (Hymenoptera: Cephidae) in China

**DOI:** 10.3390/biology14091240

**Published:** 2025-09-10

**Authors:** Meiqi Li, Jingling Li, Zehao An, Shasha Wang, Youpeng Lai

**Affiliations:** 1 Qinghai Provincial Key Laboratory of Comprehensive Management of Agricultural Pests, Qinghai University, Xining 810016, China; 13369796753@163.com (M.L.); ljl2015414116@163.com (J.L.); 15639266794@163.com (Z.A.); wangshasha2002@163.com (S.W.); 2 Plant Protection Institute, Qinghai Academy of Agricultural and Forestry Sciences, Xining 810016, China

**Keywords:** *Cephus fumipennis*, entomopathogenic fungus, isolation and identification, pathogenicity, *Clonostachys rosea*

## Abstract

*Cephus fumipennis*, a significant pest of highland spring wheat, damages crops through larval boring and feeding within wheat stalks. This activity disrupts nutrient and water transport, causing severe yield reductions. To find microbial biocontrol agents targeting this pest, primary entomopathogenic microorganisms were isolated and identified from naturally infected, deceased *C. fumipennis* larvae. Morphological examination and ITS-based phylogenetic analysis tentatively identified the isolate as the entomopathogenic fungus *Clonostachys* sp. (strain CF01). Third-instar larvae of *C. fumipennis* were inoculated with conidial suspensions of the CF01 strain at concentrations of 1 × 10^5^, 1 × 10^6^, 1 × 10^7^, and 1 × 10^8^ spores/mL. Spore suspensions of different concentrations demonstrated pathogenicity against third-instar larvae of *C. fumipennis.* The optimal growth conditions for strain CF01 were identified as follows: PPDA medium, 25 °C, fructose as the carbon source, and yeast extract as the nitrogen source. Photoperiod exhibited no significant effect on either mycelial growth or sporulation. These findings indicate that the CF01 strain possesses considerable potential for the biocontrol of *C. fumipennis*.

## Simple Summary

*Cephus fumipennis* is a significant pest of wheat crops. An entomopathogenic fungus, identified as *Clonostachys* sp., based on morphological and molecular analyses, was isolated from dead *C. fumipennis* larvae. A spore suspension derived from this isolate was used to inoculate third-instar larvae. Mycelial growth rate and sporulation served as key parameters to determine optimal growth conditions. Results demonstrated that strain CF01 is pathogenic to *C. fumipennis* larvae. As no prior studies have documented entomopathogenic fungi infecting *C. fumipennis*, these findings provide a novel approach for its biological control.

## 1. Introduction

*Cephus fumipennis* Eversmann, commonly known as the gray-winged wheat stem wasp (Hymenoptera: Cephidae), is a major pest of wheat in northwestern China. Its primary distribution encompasses Qinghai and Gansu provinces, with additional occurrences reported in Shaanxi, Henan, and Shanxi, among others [1,2,3]. In 2016, *C. fumipennis* was first documented in the Tacheng district, Xinjiang Uygur Autonomous Region. Severe infestations by this wasp cause wheat failure, white ears, reduced thousand-grain weight, and spike weight, ultimately diminishing yield and quality, with recorded losses reaching 24%. Infestation rates average 7.9% in Gansu and 12.5% in Qinghai. Notably, in Tacheng district, Xinjiang, *C. fumipennis* infests approximately 50% of spring wheat fields, with rates exceeding 70% in severely affected areas; its impact has intensified annually [4]. Consequently, *C. fumipennis* significantly threatens wheat production in northwestern China, leading to substantial economic losses.

In Qinghai province’s spring wheat-producing areas, *C. fumipennis* exhibits a univoltine life cycle. Mature larvae overwinter in diapause within thin cocoons located in wheat stubble or stem bases. Pupation occurs in April of the following year, with adult emergence and mating peaking in mid-to-late May. Using their ovipositors, female wasps saw small openings in wheat stems to deposit eggs scattered along the stem’s inner wall. Upon hatching, larvae feed extensively within the stems. At maturity, larvae migrate downward to the stem base, where they perform girdling behavior—severing vascular tissues while retaining epidermal connection—before constructing overwintering cocoons in the stubble [5,6]. Field distribution of *C. fumipennis* follows an aggregated pattern, demonstrating significant edge effects. Adults predominantly cluster near field margins and exhibit rapid migration to adjacent wheat fields, resulting in more severe infestations along field edges [7].

Integrated Pest Management (IPM) for *C. fumipennis* prioritizes resistant cultivar development combined with post-harvest deep plowing of stubble, supplemented by chemical control [8]. Nevertheless, chemical methods remain predominant in field applications. Biological control investigations identify *Collyria catoptron* Wahl as the dominant parasitoid, achieving average parasitism rates of 9.6% in Gansu and 1.6% in Qinghai [9]. For the Nearctic *Cephus cinctus*, the parasitoids *Bracon cephi* Gahan and *B. lissogaster* Muesebeck serve as key natural enemies [10,11]. The beetle *Phyllobaenus dubius* Wolcott preys upon wheat stem sawflies; however, its unconfirmed life history and limited rearing potential necessitate further evaluation of its biocontrol utility [12]. Entomopathogenic nematodes (EPNs) demonstrate efficacy against sawfly larvae and pupae, particularly when enhanced with chemical additives [13]. Pathogenic fungi studies reveal *Fusarium* spp. induce high larval mortality in *C. cinctus*-infested spring wheat [14]; five *Fusarium* species cause 90% larval mortality at high concentrations [15,16]. However, given *Fusarium*’s detrimental impact on wheat growth and yield, its practical application requires a thorough risk-benefit assessment.

Entomopathogenic fungi infect insects through cuticular penetration, proliferating within or on the host body to cause mortality. These fungi represent promising biological control agents due to their pest-specific lethality and minimal environmental impact [17,18]. They contribute significantly to agricultural pest management as key components of IPM systems [19]. Approximately 100 genera encompassing 1000 fungal species have been identified as entomopathogens, with at least 750 species demonstrating insecticidal activity [20,21,22]. Commercially deployed species include *Beauveria bassiana* (Bals.-Criv.) Vuill., *Metarhizium anisopliae* (Metschn.) Sorokin, *Paecilomyces fumosoroseus* (Wize) Kepler, B. Shrestha & Spatafora., and *Akanthomyces lecanii* (Zimm.) Spatafora, Kepler & B. Shrestha [23]. *Clonostachys* spp. are multifunctional microorganisms that play key ecological and biological control roles. They occur widely across diverse habitats, with a pronounced prevalence in soil [24]. Regarding insect pathogenicity, certain *Clonostachys* spp. can infect Hemiptera, Hymenoptera, and Coleoptera. Known insect hosts remain limited: initial documentation in 2006 identified *C. rosea* infestations causing mortality in the hemipteran *Oncometopia tucumana* [25], Lu et al. [26] isolated a highly virulent strain of *C. rosea* from larvae of *Cephalcia chuxiongica* (Hymenoptera).

In this study, diseased larvae of the gray-winged wheat stem sawfly, *C. fumipennis*, were collected from spring wheat fields. A fungal isolate obtained and purified from these larvae was tentatively identified—based on morphological and ITS sequence analyses—as *C. rosea*, and its pathogenicity to *C. fumipennis* was confirmed. The optimal culture medium and conditions for this isolate were subsequently determined. Our findings indicate that this strain is a promising candidate for the biological control of *C. fumipennis*.

## 2. Materials and Methods

### 2.1. Fungal Isolation and Purification

*Cephus fumipennis* larvae exhibiting disease-induced mortality were collected from root stubble in spring wheat fields of Tal Town, Datong County, Xining City, Qinghai Province (37°1′41″ N, 101°36′46″ E; elevation 2511.8 m). Specimens were transported to the laboratory for fungal isolation and purification.

### 2.2. Strain Isolation and Purification

Dead *C. fumipennis* larvae were maintained in sterile Petri dishes under controlled conditions (20 °C, 80% RH) until conidiogenesis occurred. Hyphae and conidia emerging on cadaver surfaces were aseptically transferred using an inoculation loop within a super-clean worktable. The fungal material was inoculated onto a potato dextrose agar plate (PDA; formulation detailed in Table 1) and incubated at 25 °C. After 7 days, a spore suspension was prepared from the plate and subjected to a series of gradient dilutions. A 100 μL aliquot of the suspension was pipetted onto the center of a culture medium plate and spread evenly. Following 3 days of additional incubation, well-isolated colonies became visible on plates with appropriate dilution densities. Distinct colonies exhibiting well-defined margins were selected with a sterile inoculation needle and transferred to fresh plates, resulting in pure single-spore isolates. Final purified isolates were preserved on PDA slants at 4 °C for long-term storage.

### 2.3. Morphological Characterization

Strain morphology was assessed by light microscopy, examining hyphal structures, conidiogenous apparatus, and conidial dimensions. Colony characteristics (pigmentation, texture, growth pattern) on PDA were documented photographically after 10 days at 25 °C. The pathogen was identified morphologically following the Manual of Fungal Identification [27].

### 2.4. Molecular Identification

Genomic DNA Extraction of Strain CF01. The ITS region was amplified with universal ITS1/ITS4 primers under the following protocol: reaction mixture (25 μL): 1 μL each primer (10 μM), 1 μL DNA template, 12.5 μL PCR MasterMix, 9.5 μL ddH_2_O; the PCR reaction program: initial denaturation at 98 °C for 3 min, 35 cycles of denaturation at 98 °C for 10 s, annealing at 55 °C for 15 s, and extension at 72 °C for 30 s/kb; final extension at 72 °C for 5 min. Amplification products were electrophoresed on 1% agarose gels, and the target amplicon was excised from the gel and submitted to Beijing Qingke Biotechnology Co., Ltd. (Beijing, China) for Sanger sequencing. Resulting sequences were aligned against the NCBI database via BLASTn. Phylogenetic reconstruction used the neighbor-joining algorithm in MEGA 7.0 with the Kimura 2-parameter model, bootstrap-validated (1000 replicates).

### 2.5. Larval Bioassay with Fungal Strains

The isolate obtained in Section 2.2 was cultured on PDA plates and incubated at 25 °C for 10 days. Conidia were harvested using 0.05% Tween-80 sterile aqueous solution and adjusted to 1 × 10^5^, 1 × 10^6^, 1 × 10^7^, and 1 × 10^8^ spores/mL. Healthy third-instar *C. fumipennis* larvae were individually reared and inoculated via immersion in conidial suspensions (15 s) across graded concentrations. After removing excess moisture with sterile filter paper, larvae were maintained at 22 ± 1 °C, 75% RH, under a 16:8 h (L:D) photoperiod. Fresh wheat straw segments were provided as the sole diet and replaced every three days to ensure food freshness. Each treatment included three replicates of 10 larvae, with 0.05% Tween-80 sterile solution serving as the negative control. Mortality was recorded daily until the 15th day. Cadavers were surface-sterilized with 75% ethanol for 10 s, incubated at high humidity (>90% RH), and monitored for signs of mycosis. Cumulative mortality and corrected mortality rates were calculated based on daily mortality counts, respectively.

$$Mortality\;rate=\frac{Total\;number\;of\;dead\;insects}{Total\;number\;of\;insects\;in\;treatments}\times 100\%$$

$$Corrected\;mortality\;rate=\frac{Treatment\;mortality\;rate-Control\;mortality\;rate}{1-Control\;mortality\;rate}\times 100\%$$

### 2.6. Optimization of the Culture Conditions of the Isolated Fungus

#### 2.6.1. Influence of Medium on Fungal Growth and Sporulation

The optimal medium for both fungal growth and sporulation of *C. rosae* strain CF01 was evaluated using six culture media (Table 1). A mycelial plug (7 mm in diameter) obtained from the actively growing margin of a fungal colony was placed at the center of each plate and incubated at 25 °C for 7 days. Colony morphology was observed daily, and the colony diameter was measured along two perpendicular diameters to calculate the average daily growth rate. All tests were repeated three times. After the incubation period, spores were harvested under sterile conditions using a 10 mL 0.05% Tween-80 solution, and a spore suspension was prepared. Sporulation yield was determined using a hemocytometer under an optical microscope.

#### 2.6.2. Optimizing the Carbon and Nitrogen Sources for Fungal Growth and Sporulation

Using PDA as the base medium, equimolar concentrations of fructose, maltose, sucrose, or soluble starch replaced glucose as carbon sources; nitrogen-equivalent amounts of (NH_4_)_2_SO_4_, NH_4_NO_3_, urea, or yeast extract replaced peptone. Mycelial plugs (7 mm in diameter) were cultured on media with different carbon and nitrogen sources at 25 °C in the dark for 7 days. All tests had three repeats. The fungal growth rate and sporulation yield were calculated as described in Section 2.6.1.

#### 2.6.3. Effect of Temperature on Fungal Growth and Sporulation

Mycelial plugs containing mycelia (d = 7 mm) were inoculated on PDA plates at 15, 20, 25, and 30 °C, as described in Section 2.6.1, and the fungal growth rate and sporulation yield were calculated. Each test was repeated three times.

#### 2.6.4. Optimization of Photoperiod for Fungal Growth and Sporulation

PDA plates inoculated with fungal isolates were incubated under three different photoperiod regimes: continuous light (24 L:0 D), alternating light/dark (12 L:12 D), and continuous darkness (0 L:24 D), all maintained at 25 °C for 7 days. The calculation of mycelial growth rate and sporulation yield is the same as in Section 2.6.1.

### 2.7. Statistical Analysis

Data were organized in Excel, SPSS 27.0 and GraphPad Prism 9.0. and used for statistical analysis. Analysis of variance (ANOVA) and least significant difference (LSD) tests were performed for fungal growth, spore production, and insect mortality rates. LT_50_, LC_50,_ and their 95% confidence limits (CI) were calculated by probit probability analysis. Graphs were prepared GraphPad Prism 9.0.

## 3. Results

### 3.1. Morphological Identification of Strains

**Figure 1:**
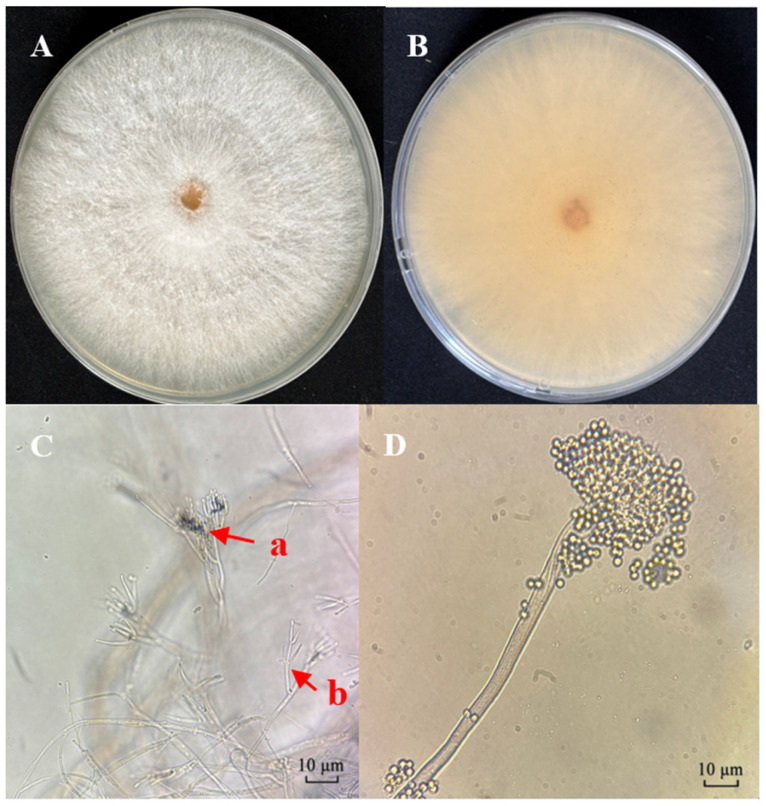
Morphological characteristics of *C. rosea* strain CF01. (**A**) Colony surface on PDA medium after 10 days. (**B**) Colony reverse on PDA medium. (**C**) (a) Penicillate-type conidiophores. (b) Verticillate-type conidiophore. (**D**) Conidia.

Morphological characteristics of the isolate were observed on PDA medium. Colonies displayed white floccose surfaces with irregular hyphal density (Figure 1A), exuding droplets; reverse sides were light yellow (Figure 1B). After 15 days of incubation, light pink conidial layers developed. Microscopic examination revealed conidiophores with Penicillate-type conidiophores and Verticillate-type conidiophores (Figure 1C) and ovate (diameter: 1.5–2.5 μm) or subglobose (3–5 μm × 2–3.5 μm) conidia; conidia adhere to one another and form aggregates (Figure 1D). Among these, the penicillate-type conidiophores are scattered and vary in length. They exhibit swollen bases and slender necks. The apical regions of the main stems branch multiple times, forming a broom-like structure measuring 9–25.5 μm × 1.5–2 μm. The verticillate-type conidiophores are symmetrically branched, slightly inflated at the base, and gradually taper toward the apex, with dimensions of 8.5–20.5 μm × 1–2 μm.

### 3.2. Molecular Biological Identification of the Strain

**Figure 2:**
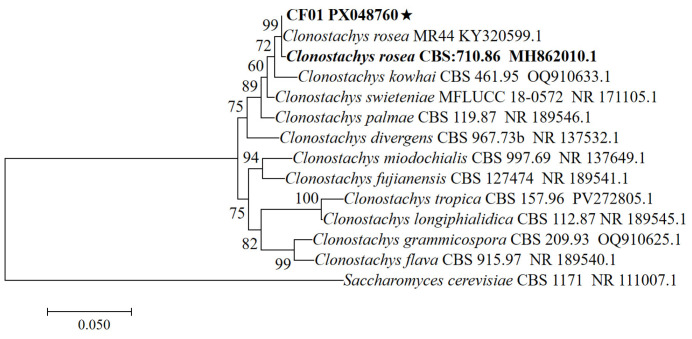
Phylogenetic analysis of the CF01 strain and related taxa based on ITS sequence alignment. Tree reconstructed using the neighbor-joining method (bootstrap values from 1000 replicates shown at nodes). The scale bar indicates 0.05 substitutions per site. MH862010 is *C. rosea*’s type strain. The asterisk denotes the CF01 strain.

Genomic DNA was extracted from the isolate. Amplification of the ITS region yielded a 576 bp amplicon. Relevant sequences were aligned, as shown in Figure 2. ITS sequences showed 99% similarity between the isolate and *C. rosea* (KY320599) as well as its type strain (MH862010). The sequences obtained in this study were submitted to GenBank with the accession number PX048760.

### 3.3. Pathogenicity of C. rosea CF01 Against C. fumipennis Larvae

**Figure 3:**
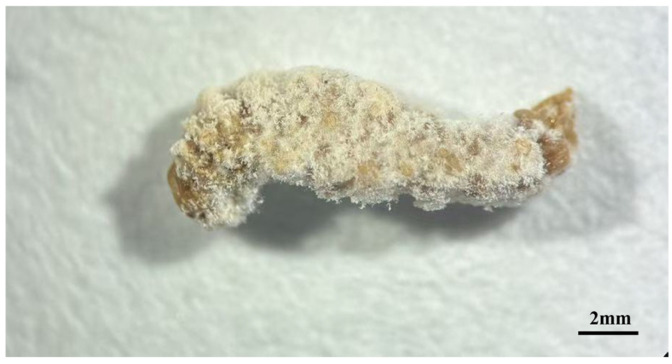
Mycosis progression in third-instar *Cephus fumipennis* larvae inoculated with CF01 strain (1 × 10^7^ spores/mL).

After larvae of *C. fumipennis* were inoculated with the CF01 strain, cadavers were collected daily, surface-disinfected, and incubated under high humidity. Mycelia first emerged from the head and intersegmental membranes, gradually enveloping the entire larva to form a mummified cadaver (Figure 3).

**Figure 4:**
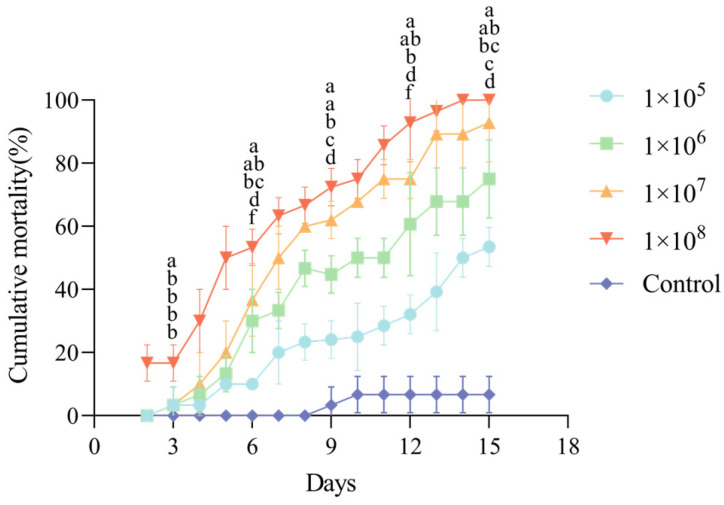
Pathogenicity of the CF01 strain on third instar larvae of the *C. fumipennis*. The mortality rate trend over time (in days) after insect treatment with four different concentrations of CF01. Data are mean ± SE. Letters on the error bars indicate significant differences analyzed using ANOVA with the LSD test (*p* < 0.05).

The corrected cumulative mortality of third-instar *C. fumipennis* larvae exposed to CF01 strain spore suspensions at different concentrations over time is shown in Figure 4. A clear dose- and time-dependent effect was observed; at every tested concentration, mortality rose gradually over time. Statistical analysis revealed that, beginning on day 4, the mortality rate in all treatment groups was significantly higher than that in the control group (*p* < 0.05).

Spore suspensions of different concentrations demonstrated pathogenicity against third-instar larvae of *C. fumipennis*. At a concentration of 1 × 10^8^ spores/mL, strain CF01 showed the fastest lethal effect, with an LT_50_ value of 5.368 days (Table 2). The LT_50_ increased as the spore concentration decreased. Table 3 presents the LC_50_ values of strain CF01 against third-instar *C. fumipennis* larvae at 7, 10, and 14 days post-infection, which decreased over time.

### 3.4. Optimization of the Culture Conditions of the Isolated Fungus

#### 3.4.1. Optimization of Culture Medium for Strain CF01

**Figure 5:**
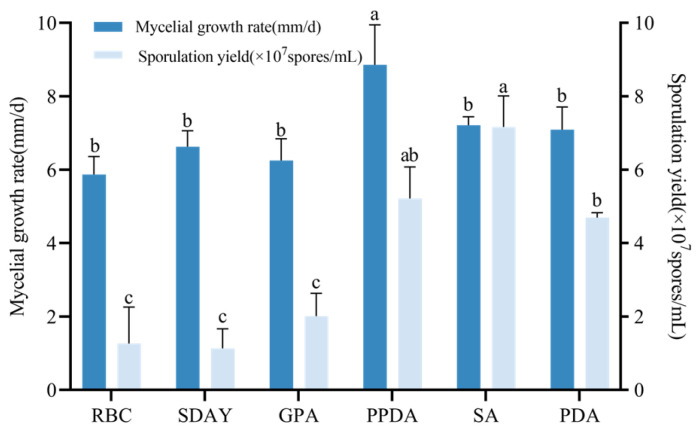
Mycelial growth rate and sporulation yield of CF01 on different media. Data are mean ± SE. Letters on the error bars indicate significant differences analyzed by ANOVA with the LSD test (*p* < 0.05).

Following 7-day incubation on varied media, strain CF01 exhibited significantly different growth and conidiation. PPDA medium yielded maximum mycelial growth, with the fastest growth rate being 8.86 mm/d (F_5,12_ = 8.203, *p* < 0.05), exceeding all other media. Conidial production peaked on SA medium (7.12 × 10^7^ spores/mL), with PPDA and PDA generating 5.22 × 10^7^ and 4.70 × 10^7^ spores/mL, respectively. Minimal sporulation occurred on RBC, SDAY, and GPA media (F_5,12_ = 35.23, *p* < 0.001) (Figure 5). Based on the analysis of growth rate and spore production, the optimal medium for the CF01 strain growth is PPDA medium.

#### 3.4.2. Effects of Temperature, Photoperiod, Carbon, and Nitrogen Sources on Growth and Sporulation of Strain CF01

**Figure 6:**
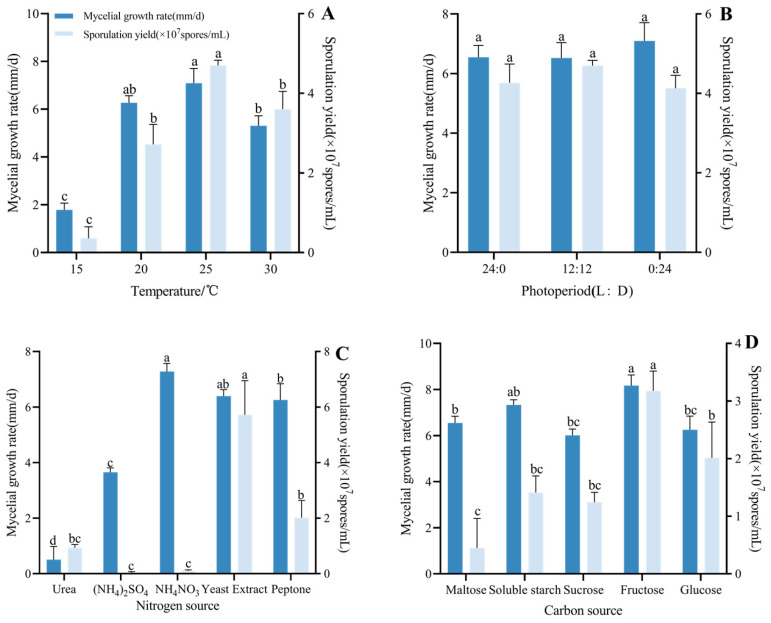
Effects of different temperatures (**A**), photoperiods (**B**), carbon (**C**), and nitrogen (**D**) sources on mycelial growth rate and sporulation yield in the CF01 strain. Data are mean ± SE. Letters on the error bars indicate significant differences analyzed by ANOVA with the LSD test (*p* < 0.05).

The CF01 strain was capable of growth across a temperature range of 15 to 30 °C. Statistical analysis revealed significant differences in both growth rates and conidial yields among the different temperature treatments (F_3,8_ = 90.57, *p* < 0.001). Optimal mycelial growth rate occurred at 25 °C (7.09 mm/d), which was not statistically different from 20 °C, while 15 °C showed a minimal growth rate (Figure 6A). Conidiation peaked at 25 °C (4.70 × 10^7^ spores/mL), significantly exceeding other temperatures (F_3,8_ = 73.80, *p* < 0.001). The strains exhibited no significant differences in growth or spore production across the different photoperiod regimes. Maximal mycelial growth rate occurred under continuous darkness (0 L:24 D), while moderate conidiation was observed at 12 L:12 D (Figure 6B).

Regarding nitrogen sources, colonies exhibited the fastest mean daily growth rate when supplied with yeast extract or NH_4_NO_3_, demonstrating no significant difference between these two sources. Ammonium nitrate supported a growth rate of 7.32 mm/d. Growth was slowest on urea-containing medium (F_4,10_ = 148.6, *p* < 0.001). Furthermore, spore production under yeast leaching powder reached 5.72 × 10^7^ spores/mL, significantly exceeding that of all other tested nitrogen sources (F_4,10_ = 43.26, *p* < 0.001). In contrast, ammonium sulfate as a nitrogen source resulted in no spore production, while ammonium nitrate yielded substantially fewer spores at 0.10 × 10^7^ spores/mL; these findings are presented in Figure 6C. Among carbon sources, colonies grew significantly faster on media containing fructose or soluble starch compared to those containing maltose or sucrose. Fructose supported the highest growth rate, attaining 8.17 mm/d (F_4,10_ = 15.22, *p* < 0.001). Spore production was also maximal with fructose, reaching 3.18 × 10^7^ spores/mL and significantly exceeding all other tested carbon sources (F_4,10_ = 17.59, *p* < 0.001) (Figure 6D). In summary, the optimal carbon source for the CF01 strain is fructose, and the optimal nitrogen source is yeast leaching powder.

## 4. Discussion

In this study, an entomopathogenic fungal strain was isolated from diseased *C. fumipennis* larvae. Based on morphological and molecular analyses, the isolate was preliminarily identified as *Clonostachys* sp. This study relied solely on ITS sequences for species identification, which represents a limitation of our work. Further multi-locus phylogenetic analysis (including genes such as TUB2 and TEF1-α) will be required in subsequent studies to clarify the taxonomic status of strain CF01. This fungus demonstrated notable pathogenicity against *C. fumipennis* larvae, suggesting its promising potential as a biological control agent. At a conidial concentration of 1 × 10^8^/mL, strain CF01 achieved the most rapid lethality, with a median lethal time (LT_50_) of 5.368 days. The experiment conducted by Lu et al. also confirmed the pathogenicity of *C. rosea* against *C. chuxiongica*, a hymenopteran insect.

Entomopathogenic fungi exhibit broad parasitic host ranges and low resistance development risk, rendering them advantageous for agricultural and forestry pest biocontrol. Despite this potential, *C. fumipennis* remains understudied regarding its pathogenic fungi. This work presents the first characterization of *C. rosea* as a larval pathogen of *C. fumipennis*. Notably, our results contrast with Lu et al. [26] regarding optimal sporulation conditions: whereas they reported ammonium nitrate and urea as maximal sporulation-promoting nitrogen sources at 25 °C with null sporulation on yeast extract powder, we observed peak spore production specifically with yeast extract powder. The data reported by Lu et al. were collected in Xundian county, Kunming city, Yunnan province, China—a region with markedly distinct climatic conditions from those at our study site. Xundian County is characterized by elevated temperatures, greater humidity, and a lower altitude in contrast to the conditions at our study site. These climatic and topographic disparities represent plausible factors contributing to the divergent outcomes between the studies. Nevertheless, the observed mycelial growth kinetics and colonial morphology align with other reports [25].

*Clonostachys rosea* (Ascomycota, Hypocreales, Bionectriaceae) is a biocontrol fungus widely distributed in Chinese soils. As an endophytic fungus, *C. rosea* parasitizes diverse hosts, including plants, insects, and nematodes [24], while suppressing phytopathogens such as *Sclerotinia sclerotiorum*, *Fusarium* spp., and *Rhizoctonia* spp. [28]. This fungus colonizes plant roots to provide sustained protection [29], enhances plant growth and seed germination [30], and induces systemic resistance [31]. Its applications target fruit/vegetable gray mold [32], root rot of soybean [33], black scurf on potato [34], barley spot blotch [35], nematodes [36,37], and arthropod pests. *C. rosea* has been documented as an entomopathogenic fungus isolated from cadavers of various insect hosts, including *Oncometopia tucumana* and *Bemisia tabaci* (Hemiptera), *C. chuxiongica* (Hymenoptera), and *Ophrida xanthospilota* (Coleoptera) [38]. Additionally, it exhibits pathogenicity against Lepidoptera pests such as the diamondback moth (*Plutella xylostella*) and Coleoptera, including the grain beetle (*Trogoderma granarium*) [39,40]. The CF01 strain investigated in this study was isolated from carcasses of deceased *C. fumipennis* larvae. This finding further expands the known host range and geographical distribution of *C. rosea* while also contributing new evidence regarding its entomopathogenic potential.

The CF01 strain isolated in this study demonstrated dual functionality: it infests *Cephus fumipennis* (wheat stem sawfly) while concurrently suppressing *Fusarium graminearum* (*Gibberella zeae*), the causal agent of wheat scab [41,42]. This fungus exhibits mycoparasitic activity against *F. graminearum* [43], providing effective wheat scab control. Its broad-spectrum inhibition of phytopathogenic fungi enables simultaneous management of wheat stem sawfly and associated fungal diseases.

Studies of plant systems confirm that endophytic fungi reduce insect survival [44,45], prompting recent efforts to exploit endophytic colonization for enhanced pest control. For instance, endophytic establishment of *Beauveria bassiana* in banana plants significantly increased larval mortality of the banana weevil *Cosmopolites sordidus* [46]. These fungi enhance pest resistance through secondary metabolite production and induce systemic resistance [47,48], while indirectly impacting insect viability via plant-mediated defense responses [49]. Given that *C. rosea* is a widely colonizing endophyte, its application could similarly improve control efficiency. Colonization strategies for wheat include foliar sprays, root immersion in spore suspensions, or seed treatments, with pre-inoculation leaf surface wounding enhancing endophytic establishment [50]. Such colonization would increase *C. fumipennis* exposure to fungal spores within wheat tissues, potentially reducing infestation rates [51]. Since *C. fumipennis* larvae reside concealed within wheat stalks—presenting control challenges—the endophytic pathway represents a promising biological control strategy.

This study confirms the pathogenicity of the CF01 strain toward *C. fumipennis* larvae. Limitations include the single-generation design, which precluded assessment of genetic and phenotypic stability across successive cycles, and without conducting field experiments, it is difficult to know whether the strain will interact with other soil microorganisms in wheat fields. Future research should therefore evaluate non-target effects on beneficial insects in wheat fields, elucidate its pathogenic mechanisms, include spore germination rate as a critical parameter, and verify whether the CF01 strain can colonize wheat plants to enhance pest-control efficacy under field conditions.

## 5. Conclusions

In this study, a fungal strain isolated from *C. fumipennis* was identified as *Clonostachys* sp. strain CF01 through integrated morphological characterization and ITS rDNA sequence analysis. These findings provide a foundation for deploying entomopathogenic fungi in biological control programs targeting *C. fumipennis* and support subsequent investigations into its pathogenic mechanisms and rhizosphere colonization dynamics in wheat.

## Figures and Tables

**Table 1:** Test media and their formula.

Medium	Medium Component
Potato Dextrose Agar (PDA)	Peeled potato 200 g/L, Dextrose 20 g/L, Agar 18 g/L, pH unadjusted
Starch Agar (SA)	soluble starch 40 g/L, yeast paste 5 g/L, Agar 20 g/L, pH unadjusted
Dextrose Peptone Agar (GPA)	Peptone 10 g/L, Dextrose 40 g/L, Agar 20 g/L, pH unadjusted
Rose Bengal Chloramphenicol Agar (RBC)	Peptone 5.0 g/L, Glucose 10.0 g/L, KH_2_PO_4_ 1.0 g/L, MgSO4 0.5 g/L, 1/3000 Rose Bengal red 100 mL/L, Agar 18.0 g/L, pH unadjusted
Peptone Potato Dextrose Agar (PPDA)	Peeled potato 200 g/L, Glucose 20 g/L, Peptone 20 g/L, Agar 18 g/L, pH unadjusted
Sabouraud Dextrose Agar with Yeast Extract (SDAY)	Glucose 40 g/L, Yeast paste 10 g/L, Peptone 10 g/L, Agar 20 g/L, pH unadjusted

**Table 2:** Virulence regression equations for LT_50_ values of *C. rosea* CF01 against *C. fumipennis*.

Concentration (Spores/mL)	Virulence Regression Equation	LT_50_ (Days)	R^2^	95%CI (Days)
1 × 10^8^	y = 3.566x − 2.603	5.368	0.869	4.356–6.307
1 × 10^7^	y = 4.500x − 3.936	7.493	0.986	6.467–8.513
1 × 10^6^	y = 3.462x − 3.427	9.768	0.976	8.343–11.792
1 × 10^5^	y = 2.860x − 3.407	15.535	0.954	12.252–27.060

**Table 3:** Virulence regression equations for LC_50_ values of *C. rosea* CF01 against *C. fumipennis*.

Days	Virulence Regression Equation	LC_50_ (Days)	R^2^	95%CI (Days)
7	y = 0.397x − 2.812	1.217 × 10^7^	0.998	7.178 × 10^5^–5.285 × 10^15^
10	y = 0.452x − 2.814	1.682 × 10^6^	0.949	9.011 × 10^3^–2.725 × 10^7^
14	y = 0.720x − 3.706	1.405 × 10^5^	0.979	4.220 × 10^2^–6.688 × 10^5^

## Data Availability

Data are contained within the article.
